# Comparing mechanical and enzymatic isolation procedures to isolate adipose‐derived stromal vascular fraction: A systematic review

**DOI:** 10.1111/wrr.13228

**Published:** 2024-10-24

**Authors:** Mustafa Uguten, Nanouk van der Sluis, Linda Vriend, J. H. Coert, Martin C. Harmsen, Berend van der Lei, Joris A. van Dongen

**Affiliations:** ^1^ Department of Plastic, Reconstructive and Hand Surgery Medical Center Leeuwarden Leeuwarden The Netherlands; ^2^ Department of Plastic, Reconstructive and Hand Surgery University Medical Center Utrecht, University of Utrecht Utrecht The Netherlands; ^3^ Department of Surgery Erasmus University Medical Center, University Medical Center Rotterdam Rotterdam The Netherlands; ^4^ Department of Pathology & Medical Biology University of Groningen and University Medical Center Groningen Groningen The Netherlands; ^5^ Department of Plastic Surgery University Medical Center Groningen, University of Groningen Groningen The Netherlands

**Keywords:** adipose stromal cells, clinical grade stromal vascular fraction, enzymatic isolation, lipoaspirate, lipografting, mechanical isolation, stromal vascular fraction

## Abstract

The stromal vascular fraction of adipose tissue has gained popularity as regenerative therapy for tissue repair. Both enzymatic and mechanical intraoperative SVF isolation procedures exist. To date, the quest for the preferred isolation procedure persists, due to the absence of standardised yield measurements and a defined clinical threshold. This systematic review is an update of the systematic review published in 2018, where guidelines were proposed to improve and standardise SVF isolation procedures. An elaborate data search in MEDLINE (PubMed), EMBASE (Ovid) and the Cochrane Central Register of Controlled Trials was conducted from September 2016 to date. A total of 26 full‐text articles met inclusion criteria, evaluating 33 isolation procedures (11 enzymatic and 22 mechanical). In general, enzymatic and mechanical SVF isolation procedures yield comparable outcomes concerning cell yield (2.3–18.0 × 10^5^ resp. 0.03–26.7 × 10^5^ cells/ml), and cell viability (70%–99% resp. 46%–97.5%), while mechanical procedures are less time consuming (8–20 min vs. 50–210 min) and cost‐efficient. However, as most studies used poorly validated outcome measures on SVF characterisation, it still remains unclear which intraoperative SVF isolation method is preferred. Future studies are recommended to implement standardised guidelines to standardise methods and improve comparability between studies.

**Abbreviations**
ADSC: Adipose‐derived stromal cells
AIS: Automated isolation system
CCD: Cell washing concentration device
cSVF: Cellular stromal vascular fraction
CYT: Cytori
DiS: Dissociation by inter‐syringe processing
EPCS: Endothelial progenitor cells
ECM: Extracellular matrix
FAT‐1 and 2: Fractionation of adipose tissue procedure 1 and 2
HT‐NANO: Hy‐tissue Nanofat
HYTISSUE: Hy Tissue SVF
IFATS: International Federation of Adipose Tissue Therapeutics
ISCT: International Society of Cellular Therapy
LCN: LipocubeNano
LGSVF: LG SVF isolation
LIPOG: Lipogems
LIPOK: Lipokit system
MLYZER: Microlyzer
NANO: Nanofat
NANO2: Nano fat 2.0
NANOT: Nanotranfer
NANOT2: Nanotransfer 2.0
PRISMA: Preferred Reporting Items for Systematic Reviews and Meta‐Analyses
PURE: Puregraft
RBs: Rotating blades system
RIGA: Rigenera
SDUO: Stempress with duografter II
SHUF20,30, and 40: Shuffling 20, 30 and 40 times
SVF: Stromal vascular fraction
SVFG: SVF gel
tSVF: Tissue stromal vascular fraction
V/C: Vortexing and centrifugation

## 1 INTRODUCTION

Autologous fat grafting has become a popular regenerative treatment to repair tissue damage caused by, for example, surgery, trauma or fibroproliferative or congenital disorders.^1^, ^2^ This regenerative potential can be primarily attributed to the stromal vascular fraction (SVF) containing fibroblasts, adipose tissue‐derived stromal cells (ASCs), vasculature, extracellular matrix (ECM) and ECM‐bound trophic factors.^3^, ^4^ Theoretically, isolated SVF harbours a larger regenerative potential compared to regular autologous fat grafting due to the increased number of regenerative cells per millilitre.

The gold standard is the use of collagenase to isolate SVF from adipose tissue or lipoaspirate, which yields an SVF with single cells (cellular SVF (cSVF)) containing all non‐adipocyte cell types such as endothelial cells, leukocytes, pericytes and fibroblasts.^5^ However, practical application of enzymatically isolated cSVF in a clinical setting is time‐consuming and costly, while after injection cells rapidly vacate the premises.^6^ Hence, alternative methods have been developed leading to the advent of mechanical isolation procedures, which use shear stress to disrupt lipoaspirate and subsequently isolate SVF.^7^ In contrast to enzymatic isolation, mechanical isolation yields both cSVF as well as a more tissue‐like SVF (tSVF). The type of SVF isolated depends on the fraction that is used. Most mechanical isolation procedures of SVF use centrifugation to separate a pellet fraction from a floating middle layer. The pellet fraction consists of both SVF cells (cSVF) and cell debris as well as erythrocytes. The middle fraction contains tSVF consisting of the same cell types as compared to cSVF. Yet, in tSVF, most intercellular connections are preserved including cell–ECM adhesions.^8^, ^9^

Our first systematic review, in 2018, revealed that none of the enzymatic or mechanical isolation procedures could be designated as superior in terms of cell yield, viability and SVF composition. Yet, mechanical isolation proved to be more time‐efficient and less expensive compared to enzymatic isolation.^6^ Moreover, many of the included isolation procedures lacked sound scientific validation. None of the included isolation procedures fully verified their procedure according to the published validation guidelines by the International Society of Cellular Therapy (ISCT) and International Federation of Adipose Tissue Therapeutics (IFATS). Hence, van Dongen et al. proposed a few changes to the aforementioned guidelines: all validation studies should use centrifuged lipoaspirate to determine the actual volume of adipose tissue to be used for validation and cell viability of tSVF should be tested directly on tSVF instead of using additional collagenase treatment first.

In the past years, multiple new isolation procedures of clinical grade SVF have been developed and validated. The objective of this systematic review is to assess the efficacy of newly developed intraoperative isolation procedures for clinical grade SVF. This systematic review is an update of the previous from 2018.

## 2 MATERIALS AND METHODS

### 2.1 Protocol and registration

The Preferred Reporting Items for Systematic Reviews and Meta‐Analyses (PRISMA) guidelines were used to perform this study.^10^ The search strategy for this systematic review was based on a Population, Intervention, Comparison, and Outcome (PICO) framework^11^ of the former study by van Dongen et al.^6^ The study was not registered.

### 2.2 Eligibility criteria

Eligible studies were clinical or observational studies that evaluated at least two distinct intraoperative isolation procedures or one intraoperative isolation procedure in combination with a non‐intraoperative isolation protocol using human adipose tissue to isolate SVF. Studies that exclusively evaluated centrifugation forces, sonication, or the use of red blood cell lysis buffer were excluded, as were studies that focused on processing methods for fat grafting. Animal studies, case reports, conference abstracts, case series and (systematic) reviews were also excluded. The search was limited by publication date (starting from September 2016 to October 2023) and not by publication status nor language.

### 2.3 Information sources and search

A systematic literature search was conducted in the online medical databases PubMed, EMBASE (Ovid) and the Cochrane Central Register of Controlled Trials from September 2016 to October 2023. The search was limited to human studies and comprised a combination of key terms related to three distinct components (Table S1): (P) adipose tissue, adipocytes, fat, lipoaspirate with (I) cell separation, isolat*, dissociat*, concentrat*, digest*, obtained and (C) stem cells, stromal cells, autologous progenitor cells, stromal vascular, regenerative cell or vascular stroma. Reference lists of included studies were analysed to identify relevant studies missed in the searches (Table S1).

### 2.4 Study selection and data collection process

Three reviewers (M. U., L. V. and N. S.) independently assessed titles, abstracts and full texts. In the event of a disagreement during consensus meetings, a senior author (J. A. D.) gave a binding verdict.

### 2.5 Data extraction

All data were extracted by the same reviewers and consisted of the following: cell yield, cell viability, SVF composition and the duration, costs and characteristics of intraoperative isolation procedures. Effect sizes were calculated for cell yield and viability in studies that compared intraoperative isolation procedures to regular non‐intraoperative isolation protocols. Variations in the harvesting procedure were not considered in the analysis. A difference was reported when there was a statistically significant difference (*p* < 0.05).

### 2.6 Risk of bias in individual studies

Detailed demographics of study populations were obtained to represent possible confounding factors on the characteristics of ASC or SVF.

### 2.7 Summary measurements

Effect sizes were calculated for the outcome variables of cell yield and percentage of viable cells from cSVF comparing (enzymatic) intraoperative isolation procedures to non‐intraoperative isolation procedures (gold standard). To calculate effect sizes, the difference in mean outcomes between enzymatic intraoperative isolation procedures and Gold standard was divided by the standard deviation (SD) of the Gold standard. The effect size of cell yield in studies focusing mechanical intraoperative isolation procedures were not taken into account because of different start volume of lipoaspirate and end volumes of tSVF. Only studies that presented results as median value with SD were included in the analysis (Table S2).

### 2.8 Synthesis of results

In some studies, data were derived from tables and graphs when numerical values of outcomes were not provided. In case studies that did not report a name for the isolation procedures, the procedure was named enzym‐1 or mechanical‐1 in numerical order. CD marker expressions were reported to distinguish and quantify different cell types within SVF. SVF composition comparisons were made between studies and comparisons were made between intraoperative procedures with their respective controls. Controls used in studies were non‐intraoperative protocols or intra‐operative protocols or procedures. Studies that evaluated expression of a single CD marker to characterise different cell types were considered insufficient and were excluded from the analysis. Cells were divided in CD45^neg^ (adipose tissue‐derived) and CD45^pos^ cells (leukocytes), to analyse the expression of stromal cells, pericytes, vascular endothelial cells, endothelial progenitor cells, endothelial cells, lymphocytes, leucocytes and haematopoietic stem cells. The CD marker combination in this systematic review was based on the predefined combinations by van Dongen et al. (Table S4A).^6^ The CD34^pos^/CD146^pos^ population was excluded from the analysis due to the inability to discriminate between progenitor pericytes and progenitor endothelial cells.^12^

### 2.9 Risk of bias across studies

There is a potential risk of publication bias towards positive results in studies where authors have benefits in the investigated products. Disclosure agreements were reviewed to assess any potential bias. Moreover, some studies might not fully characterise the isolated SVF according to the IFATS/ISCT guidelines, which potentially resulted in biased results. Hence, an overview of the characterisation procedures according to the IFATS/ISCT guidelines is presented in 2.10.

### 2.10 Modified IFATS/ISCT index score for the measurement of adipose tissue‐derived stromal vascular fraction

The assessment of quality in each included study was evaluated using a modified IFATS and ISCT guideline as previously used in the first systematic review.^13^ If studies performed one of the following characterisation methods of SVF cells and ASCs, one point was given by the authors (M. U. and N. S.) for each of the following performed characterisations: viability of cells, flow cytometry of SVF cells, flow cytometry of ASCs (CD13, CD29, CD31, CD34, CD44, CD45, CD73, CD90, CD105, CD235a), proliferation and frequency (CFU‐F) and functional assays (adipogenic, osteogenic and chondrogenic differentiation assays) of ASCs. The maximum score for the flow cytometry of cultured ASCs and functional assays was divided by the total number of CD markers or differentiation assays possible. The maximum score in case of a full characterisation was 5.

## 3 RESULTS

### 3.1 Study selection

**FIGURE 1:**
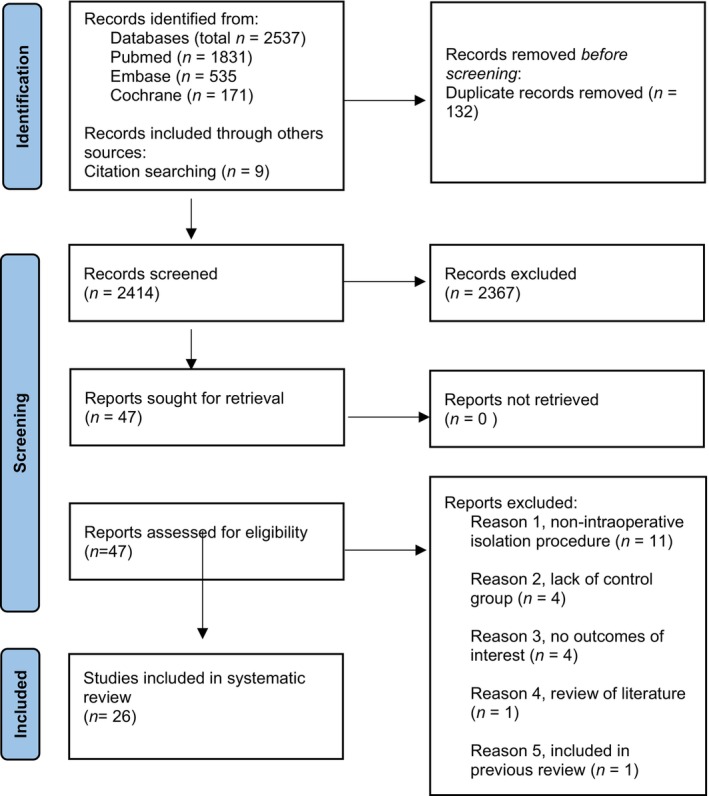
Flow diagram of study selection.

The initial database search yielded 2414 studies, of which 2367 were excluded after title and abstract screening. Forty‐seven full‐text studies were assessed on eligibility criteria. Eligible studies were excluded for the following reasons: 11 studies used a non‐intraoperative isolation method,^14^, ^15^, ^16^, ^17^, ^18^, ^19^, ^20^, ^21^, ^22^, ^23^, ^24^ 4 studies lacked the use of a control group,^23^, ^25^, ^26^, ^27^ 4 studies described isolation protocols but gave no results of interest (i.e., no SVF composition, cell yield or viability),^28^, ^29^, ^30^ 1 study concerned a review of the literature,^31^ 1 study was included in previous review by van Dongen et al.^32^ and 9 studies were identified through other sources. In total, 26 studies with 33 different intraoperative isolation procedures were included (Figure 1).^33^, ^34^, ^35^, ^36^, ^37^, ^38^, ^39^, ^40^, ^41^, ^42^, ^43^, ^44^, ^45^, ^46^, ^47^, ^48^, ^49^, ^50^, ^51^, ^52^, ^53^, ^54^, ^55^, ^56^, ^57^

### 3.2 Study characteristics

In total, 352 participants were enrolled in all studies (Table S2). The mean age of included participants was 42 years, range 21–71 years and 89% of all patients were female. Body Mass Index (BMI) ranged from 24 to 38 kg/m^2^. All included studies performed elective liposuction for aesthetic purposes. The method of infiltration and liposuction was described in nine studies. Tumescent infiltration was used prior to liposuction in five‐teen studies and the most common donor site was the abdomen (*n* = 15). Data pooling and meta‐analysis was not possible due to heterogeneity across studies in terms of methodological characteristics, for example, isolation protocols, assessment tools and analysis of SVF cells.

### 3.3 Characteristics of intraoperative isolation procedures

Intraoperative isolation procedures were divided into enzymatic (*n* = 11) and mechanical (*n* = 22) procedures (Tables 1 and 2). The enzymatic isolation protocols were mostly closed‐system devices (*n* = 10) while the mechanical isolation protocols were mostly open system (*n* = 13). Four studies centrifuged the freshly harvested adipose tissue prior to processing.^50^, ^51^, ^53^, ^54^ Other studies did not report the actual volume of lipoaspirate that was used for validation of the isolation procedures. This is relevant due to the influence on ultimate cell yield. Four enzymatic isolation procedures were modifications of previously developed intraoperative isolation protocols: LG SVF, Puregraft, Automated Isolation System (AIS), enzyme‐1.^33^, ^37^, ^38^, ^40^ GID SVF1 and GID SVF2 (GID group, Louiseville CO) both are identical intraoperative enzymatic isolation procedures, only the processed volume is different respectively 300 versus 125 mL.^33^ Multiple included mechanical isolation procedures were comparable with each other with only small modifications: SVF gel (SVFG) is based on the fractionation of adipose tissue (FAT) procedure, that is, centrifugation, shuffling through a connector and centrifugation in consecutive order. The Nanofat (NANO) procedures are based on shuffling adipose tissue through a connector as well, but do not use a prior centrifugation step. LipocubeNano (LCN) used different fractions of adipose tissue after centrifugation and shuffling through the Lipocube device, combining cSVF with tSVF.^58^ GID SVF 1, Rigenera (RIGA), LCN, Nanotransfer (NANOT), Nanofat and Lipogem (LIPOG) procedures were reported in multiple studies.^33^, ^34^, ^36^, ^42^, ^43^, ^45^, ^47^, ^48^, ^49^, ^52^, ^53^, ^56^, ^58^

**TABLE 1:** Characteristics of all enzymatic intraoperative isolation procedures.

Name	Author	Automatic/manual/semi (A/M/S)	Open/closed (O/C)	Isolation details		Time (min)	Disposable (*D*)/reusable (*R*) cost (US$)	Total cost (US$)^a^	Volume processed (ml)	Capacity (ml)	End volume (ml)	Maximum volume processed/maximum end volume (ml)
AIS	Hahn et al., 2018	A	C	Digestion, heating and agitation, centrifugation 379 *g*/5 min	Collagenase	50	‐	‐	50–100	‐	70	‐
CCD	Hayashi et al., 2021	S	C	Digestion and shaking 30 min, filtration by gravity (hollow fibre membrane module), washing	Collagenase	120	‐	‐	20–200	‐	20	‐
CYT	Francois et al., 2020	A	C	Digestion and agitation	Celase	‐	‐	‐	>100	‐	‐	‐
GID‐SVF 1	Brown et al., 2017	M	C	Digestion, filtration, centrifugation 800 *g*/10 min	GIDzyme‐2	70	‐	‐	‐	300	Pellet	‐
	Rodrigues et al., 2017.	M	C			90	D500	‐	‐	300	Pellet	‐
	Sese et al., 2019	M	C			‐	‐	‐	200	‐	Pellet	‐
GID‐SVF 2	Brown et al., 2017	M	C	Digestion, filtration, centrifugation 800 *g*/10 min	GIDzyme‐2	70	‐	‐	‐	125	Pellet	‐
LGSVF	Francois et al., 2020	S	C	Heating and agitation with orbital shaker 45 min., centrifugation 400 *g*/4 min, digestion, filtration 200 μm	Collagenase	210	D1600	5000	100	‐	15	‐
LIPOK	Raposio et al., 2016	S	C	Centrifugation 1600 *g*/6 min, digestion, centrifugation 400 *g*/4 min	Collagenase NB6	80	‐	‐	100	‐	Pellet	‐
Enzym‐1	Nürnberger et al., 2019	M	O	Digestion under shaking 180 rpm/1 h, centrifugation 1200 *g*/7 min and 300 *g*/5 min	Collagenase NB6 0.1 U/mL	82	‐	‐	100	‐	10–15	‐
PURE	Rodriques et al., 2017	M	C	Digestion under shaking, centrifugation 300 *g*/5 min	Collagenase NB6 0.1 U/mL	100	D250	‐	250	‐	Pellet	‐
SDUO	Rodriques et al., 2017	M	C	Digestion under shaking 45 min. centrifugation 300 *g*/5 min. filtration 70 μm	Collagenase NB6 0.1 U/mL	110	‐	‐	200	‐	Pellet	‐
TMI	Winnier et al., 2019	A	C	Digestion, repetitive acceleration/deceleration 30 min. filtration 200 μm, centrifugation 600 *g*/5 min twice	Matrase Reagent	55	‐	‐	25	‐	3	‐

Abbreviations: AIS, automated isolation system (Cellunit); CCD, cell washing concentration device; CYT, Cytori (Cellution system enzymatic); GID SVF 1 and GID SVF 2, GID Europe; LG SVF, isolation (Puregraft); LIPOK, Lipokit system (Medi‐khan); Enzym‐1, enzymatic isolation; PURE, Puregraft (Eurosillicone); SDUO, Stempress with Duografter II (Proteal); TMI, transpose RT/matrase isolation (InGeneron).

^a^ Total cost includes harvesting and extraction, facility and labour cost and biological quality control.

#### 3.3.1 Start volume versus end product

Enzymatic isolation procedures AIS, enzyme‐1 and LG SVF protocol processed 100 mL of fat and resulted in 70 mL, 15 mL and 10–15 mL of end volume of cSVF, respectively. This suggests an inefficient digestion of fat.^37^, ^38^, ^40^, ^44^

Of the mechanical isolation procedures, the most efficient mechanical dissociation was seen in the FAT‐1 and FAT‐2 procedure with 9.09‐fold volume reduction (Table 2).^50^ In comparison, the Nanofat procedure, Lipogem and SVF‐gel resulted in a 1.22–2.0‐fold, 2.0–3.0‐fold and 5‐fold volume reduction, respectively.^47^, ^48^, ^56^ Most other mechanical isolation procedures (*n* = 10) focused on the pellet fraction, that is, cSVF of processed lipoaspirate (Table 2). All other mechanical intraoperative isolation procedures (*n* = 12) did not report start and/or end volumes of lipoaspirate and SVF (Tables 2).

**TABLE 2:** Characteristics of all mechanical intraoperative isolation procedures.

Name	Author	Automatic/manual/semi (A/M/S)	Open/closed (O/C)	Isolation details	Time (min)	Disposable (*D*)/reusable (*R*) cost (US$)	Total cost (US$)^a^	Volume processed (mL)	Capacity (mL)	End volume (mL)	Maximum volume processed/maximum end volume (mL)
DiS	Chaput et al., 2016	M	O	Shuffling through a connector 30 times, filtering 100 μm and centrifugation 558 *g*/10 min	‐	‐	‐	10	‐	Pellet	‐
FAT‐1	Van Dongen et al., 2020	M	O	960 *g*/2.5 min centrifugation, shuffling through a three‐hole 1.4 mm connector and 960 *g*/2.5 min centrifugation	8–10	R	‐	10	10	1.1	9.09
FAT‐2	Van Dongen et al., 2020	M	O	960 *g*/2.5 min centrifugation, shuffling through a one‐hole 1.4 mm connector and 960 *g*/2.5 min centrifugation	8–10	D	‐	10	10	1.1	9.09
HT‐NANO	Quintero Sierre et al., 2023	M	O	Shuffling through a 3 mm connector 30 times and filtering 120 μm	‐	D	‐	10	10	‐	‐
HYTISSUE	Busato et al., 2020	M	C	Filtration 120 μm mesh, mechanical disaggregation by plastic rod and centrifugation 400 *g*/10 min	15–20	D	‐	30	‐	Pellet	‐
LCN	Tiryaki et al., 2020	M	O	Decantation, shuffling through lipocube in 3 different portals (resp. 1000 μm and two times 750 μm) 10 times and one time through a portal 500 μm	‐	‐	‐	20	‐	Pellet	‐
	Cohen et al., 2019	M	O		‐	D	‐	10	‐	‐	‐
	Tiryaki et al., 2022	M	O		‐	‐	‐	20	‐	‐	‐
LIPOG^b^	Senesi et al., 2019	M	C	Filtration, decantation, stainless steel marbles to mix layers (oil, adipose tissue, blood, saline), decantation, reversing devices and filtration	20	‐	‐	‐	‐	10	‐
	Vezzani et al.	M	C		‐	‐	‐	60	‐	20–30	3.0–2.0
	Cicione et al., 2023	M	C		‐	‐	‐	50	‐	‐	‐
MLYZER	Yaylaci et al., 2023	M	C	Decantation, centrifugation 1500 *g*/8 min, 31 times passing through 2400 μm and 1200 μm blade system, 100 times through 600 μm, washing and centrifugation 400 *g*/10 min	‐	‐	‐	10	‐	Pellet	‐
NANO	Lo Furno et al., 2017	M	O	Shuffling through a connector 30 times, filtration 0.6–0.4 mm mesh	‐	‐	‐	‐	‐	‐	‐
	Cicione et al., 2023	M	O	Centrifugation 1200 *g*/3 min, shuffling through connector 30 times and centrifugation 1200 *g*/3 min	‐	‐	‐	10	‐	5	2
SHUF20	Girard et al., 2022	M	O	Shuffling through Luer‐Lok connector 20 times	‐	‐	‐	10	‐	‐	‐
SHUF30		M	O	Shuffling through Luer‐Lok connector 30 times	‐	‐	‐	10	‐	‐	‐
SHUF40		M	O	Shuffling through Luer‐Lok connector 40 times	‐	‐	‐	10	‐	‐	‐
NANOT	Cohen et al., 2019	M	O	Decantation, shuffling through an one hole 2.4 mm connector 30 times, an one hole 1.2 mm 30 times and filtration 0.6–0.4 mm mesh	‐	‐	‐	10	‐	‐	‐
	Sese et al., 2019	M	O		‐	‐	‐	20	‐	‐	‐
	Yang et al., 2021^c^	M	O	Shuffling through an one hole 2.4 mm connector, filtration 0.5 mm	‐	‐	‐	10	‐	8.2	1.22
	Ramaut et al., 2023	M	O	Shuffling through an one hole 2.4 mm connector 10 times, an one hole 1.4 mm 10 times and an one hole 1.2 mm 10 times, filtration through 500 μm mesh	‐	‐	‐	10	‐	Pellet	‐
NANO2	Lo Furno et al., 2017	M	O	Shuffling through a connector 30 times only	‐	‐	‐	‐	‐	‐	‐
NANOT2	Ramaut et al., 2023	M	O	Shuffling through an one hole 2.4 mm connector 10 times, an one hole 1.4 mm 10 times and an one hole 1.2 mm 10 times, no filtration	‐	‐	‐	10	‐	Pellet	‐
RBs	Solodeev et al., 2023	M	C	Mixing with prewarmed (37°) saline, mechanically disruption by rotating bladed with an external actuator, centrifugation 400*g*/15 min, sedimentation, passing through 100 μm stainer	15	D	‐	>100	‐	‐	‐
RIGA45	De Franesco et al., 2018	A	C	Decantation, mechanical disaggregation by ceramic blade and filtration 80 μm	‐	‐	‐	‐	‐	Pellet	‐
RIGA30		A	C		‐	‐	‐	‐	‐	Pellet	‐
RIGA	Senesi et al., 2019	A	C		‐	‐	‐	‐	‐	10	‐
SVFG	Yang et al., 2021^c^	M	O	Centrifugation 1200*g*/3 min, shuffling through an one‐hole 2.4 mm connector 8 times, centrifugation 2000*g*/3 min	‐	‐	‐	10	‐	2	5
V/C	Chaput et al., 2016	M	O	Vibrating shaker 3200 vib/min, centrifugation 558 *g*/10 min	<20	‐	‐	80	‐	Pellet	‐
V/C2	Raposio et al., 2017	M	O	Vibrating shaker 6000 vib/min, centrifugation 1200 *g*/6 min	15	‐	‐	80	‐	Pellet	‐

Abbreviations: DiS, Dissociation by inter‐Syringe processing; FAT‐1 and 2, Fractionation of Adipose Tissue procedure with three‐hole connector and one‐hole connector respectively; HT‐NANO, HyTissue Nanofat; HYTISSUE, Hytissue; LCN, LipoCubeNano; LIPOG, Lipogem; MLYZER, Microlyzer (T‐biotechnology); NANO, Nanofat procedure; NANOT, NanoTransfer; NANO2, Nanofat 2.0 procedure; NANOT2, NanoTransfer without filtration; RBs, rotating blades system; RIGA45, Rigenera 45 s; RIGA30, Rigenera 30s; RIGA, Rigenera; SHUF20, 30 and 40, shuffling 20, 30 and 40 times, respectively; SVFG, SVF gel; V/C, vortexing and centrifugation; V/C2, vortexing and centrifugation 2.

^a^ Total cost includes harvesting and extraction, facility and labour cost and biological quality control.

^b^ Isolation details were described from its original protocol.

^c^ No exact data described in text; data extracted from figures.

#### 3.3.2 Duration and costs

The duration of respectively mechanical and enzymatic isolation procedures ranged from 8 to 20 min and 50 to 210 min, respectively (Tables 1 and 2), after obtaining the lipoaspirate. The duration of the enzymatic LG SVF isolation procedure was longest with 210 min.^37^ The FAT procedure was the fastest reported procedure with 8–10 min.^41^ In general, mechanical intraoperative isolation procedures yielded SVF faster than enzymatic procedures.

Studies only reported the purchase price of enzymatic intraoperative isolation procedures: GID SVF $500, LGSVF protocol $1600 and Puregraft $250, with only LG SVF protocol reporting the overall cost of $5000.^33^, ^37^, ^42^ No purchase prices of mechanical intraoperative isolation procedures were provided (Tables 1 and 2).

### 3.4 Cell yield

**FIGURE 2:**
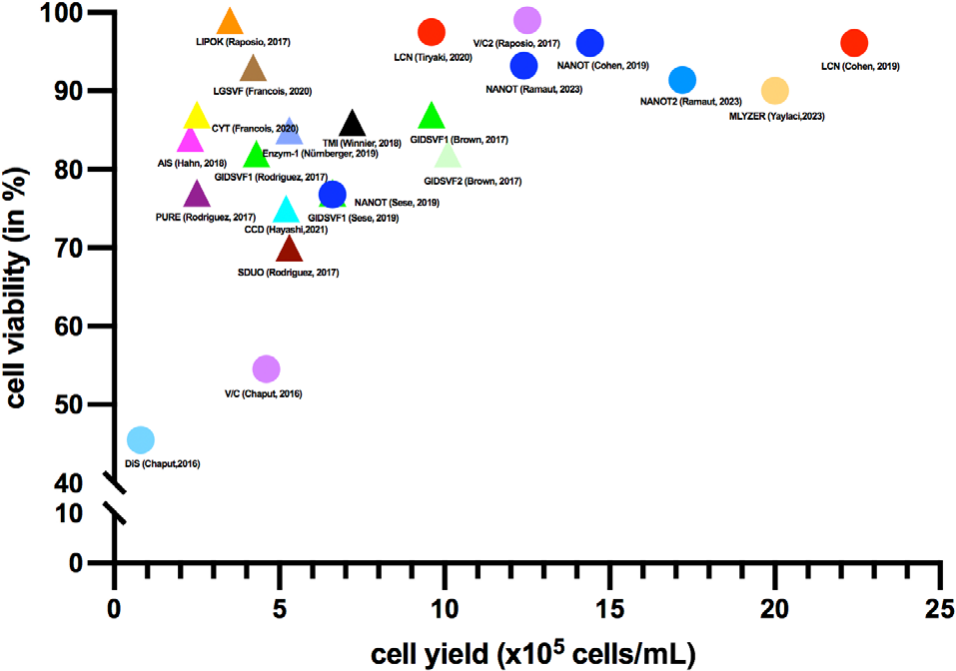
Scatter graph of all studies and the cell yield and viability of each different isolation procedure. Only studies that reported both cell yield and viability were shown.

The cell yield of 25 different isolation protocols were reported in 21 studies and ranged from 2.3 × 10^5^ to 18.0 × 10^5^ cells/mL in the enzymatic isolation procedures and from 0.3 × 10^4^ to 26.7 × 10^5^ cells/mL in mechanical isolation procedures (Figure 2 and Table S3A,B).^33^, ^34^, ^35^, ^37^, ^38^, ^40^, ^41^, ^42^, ^44^, ^45^, ^46^, ^47^, ^48^, ^49^, ^50^, ^51^, ^53^, ^54^, ^57^, ^58^, ^59^ Of the enzymatic isolation procedures, the highest cell yield was seen in the GID SVF1 and 2 devices (9.6 × 10^5^ vs. 10.1 × 10^5^ cells/mL), and the lowest in the AIS device (2.3 × 10^5^ cells/mL) (Table S3A).^33^, ^38^ One study found no difference in cell yield between GID SVF1, Puregraft and Stempress with duografter II with a mean of 4.3 × 10^5^, 2.5 × 10^5^ and 5.3 × 10^5^ cells/mL, respectively.^42^ When comparing the cell yield of the same isolation procedures among different studies, the GID SVF 1 and 2 yielded different number of cells, 9.6 × 10^5^ ± 2.1 cells/mL on average^33^ and 4.3 × 10^5^ ± 0.4^42^ on average, respectively. This is probably caused by inter‐donor variability because comparable cell yield were obtained when the same donors were used.^33^

**TABLE 3:** Effect size of studies evaluating cell yield in enzymatic isolation procedures.

Study	Enzymatic isolation procedure			Non‐intraoperative isolation protocol			Effect size
	*n*	Cell yield (×10^5^ cells)	SD	*n*	Cell yield (×10^5^ cells)	SD	
CCD, Hayashi, 2021	4	5.1	1.7	4	6.0	3.2	−0.28
GIDSVF1, Rodriguez, 2017	3	4.3	0.4	3	7.9	2.3	−1.57
Enzym‐1, Nürnberger, 2017	8	5.3	2.0	8	2.8	2.2	1.13
PURE, Rodriguez, 2017	3	2.5	0.7	3	7.9	2.3	−2.35
SDUO, Rodriguez, 2017	3	5.3	2.1	3	7.9	2.3	−1.00
TMI, Winnier, 2018^a^	12	7.2	0.1	12	0.8	0.1	−64.0

Abbreviations: CCD, cell washing concentration device; Enzym‐1, enzymatic isolation; GID SVF1, GID Europe; PURE, puregraft (Eurosilicone); SDUO, stempress with duografter II (Proteal); TMI, transpose RT/matrase isolation (InGeneron).

^a^ The comparison with isolation protocol no matrase reagent as the gold standard.

Of the mechanical isolation procedures, the FAT‐2 procedure resulted in the highest cell yield (26.7 × 10^5^ cells/mL).^50^ The lowest cell yield was seen in the Lipogem procedure (3.0 × 10^3^).^47^ Both LipocubeNano and Nanotranfer procedure were evaluated in different studies and resulted in 9.6 × 10^5^ versus 19 × 10^5^ versus 22.4 × 10^5^ and 0.4 × 10^5^ versus 6.6 × 10^5^ versus 12.4 × 10^5^ versus 14.4 × 10^5^, respectively.^35^, ^48^, ^49^, ^53^, ^58^ However, when comparing both procedures in the same study, no differences were reported.^35^

Four studies compared the same lipoaspirates obtained from their intraoperative isolation procedure to the enzymatic reference isolation protocol (gold standard) (Table 3).^33^, ^40^, ^44^, ^54^ The enzyme‐1 protocol isolated a significantly higher cell yield compared to a non‐intraoperative enzymatic isolation protocol (effect size 1.13).^40^ GID SVF1, Puregraft and Stempress with Duografter II, all resulted in a lower cell yield (effect size −1.57, −2.35 and −1.00, respectively). The most negative effect size was observed after the TMI procedure.^44^

### 3.5 Viability of nucleated cells

The viability of nucleated cells was reported in 14 studies (Figure 2) (Table S3A,B).^33^, ^34^, ^35^, ^37^, ^38^, ^40^, ^41^, ^42^, ^44^, ^45^, ^49^, ^51^, ^53^, ^54^ In enzymatic intraoperative isolation procedures, the viability ranged from 70% to 99%, with the Lipogem procedure scoring the highest percentage of viability of 99% (Table S3). The viability of nucleated cells varied largely in the mechanical isolation procedures from 46% in the Nanofat procedure to 97.5% in the LipocubeNano procedure (Table S3B).

**TABLE 4:** Effect size of studies evaluating viable nucleated cells.

Study	Procedure			Non‐intraoperative isolation protocol			Effect size
	*n*	% viable cells	SD	*n*	% viable cells	SD	
*Enzymatic*							
CCD, Hayashi, 2021	4	75.3	2.4	4	85.2	3.4	−2.91
GIDSVF1, Rodriguez, 2017	3	75.8	4.3	3	69.3	2.4	2.71
Enzym‐1, Nürnberger, 2017^A^	8	85.0	8.0	15	76.0	7.0	1.29
PURE, Rodriguez, 2017	3	81.5	1.4	3	69.3	2.4	5.08
SDUO, Rodriguez, 2017	3	77.5	1.1	3	69.3	2.4	3.42
TMI, Winnier, 2018	12	85.9	1.1	12	61.7	2.6	9.31
*Mechanical*							
DiS, Chaput, 2016	21	45.5	3.5	21	90.7	2.7	−16.7
V/C, Chaput, 2016	21	54.5	7.5	21	90.7	2.7	−13.4

Abbreviations; CCD, cell washing concentration device; DiS, dissociation by inter‐syringe processing; Enzym‐1, enzymatic isolation; GID SVF1, GID Europe; PURE, puregraft (Eurosilicone); SDUO, stempress with duografter II (Proteal); TMI, transpose RT/matrase isolation (InGeneron); V/C, vortexing and centrifugation.

When comparing the viability of both enzymatic and mechanical isolation procedures with a reference isolation protocol (gold standard), all enzymatic isolation procedures had a positive effect size and scored a higher percentage of viable nucleated cells in SVF (Table 4).

### 3.6 Composition of stromal vascular fraction

Twelve studies assessed the composition of SVF^33^, ^34^, ^35^, ^37^, ^39^, ^40^, ^41^, ^42^, ^50^, ^54^, ^55^, ^58^ (Table S4A,B). Seven studies used predefined combinations of CD marker expression by van Dongen et al. to characterise SVF composition.^33^, ^34^, ^37^, ^40^, ^48^, ^50^, ^54^, ^55^ The highest stromal cell population (CD31min/CD34pos) was reported in the enzymatic CCD procedure (52%),^54^ while the mechanical isolation procedure, Nanofat and Rotating blades system (RBs) isolated 38.1% and 22.7% stromal cells, respectively. GID SVF1 and GID SVF2 isolated 9% and 7% endothelial progenitor cells (CD31pos/CD34pos), respectively. The population of endothelial progenitor cells was the highest in enzyme‐1 (39%) and the lowest in the Nanofat procedure (0.2%).

Seven other isolation protocols used different combinations of CD markers the determine cell composition in SVF.^34^, ^35^, ^37^, ^40^, ^47^, ^49^, ^58^ Enzyme‐1 isolated significantly more ASCs (CD34pos/CD90pos) as compared to their reference protocol (40). Two studies used CD34pos/CD90pos/CD146min to determine ASCs. SVF isolated by the following procedures: Nanofat, LG SVF and Cytori contained 38%, 40% and 44% of ASCs, respectively.^34^, ^37^ In both studies, the pericyte population was based on CD45min/CD34min/CD146pos and CD90pos/CD146pos, representing 36% in the enzyme‐1, 12% in LG SVF and 10% Cytori, respectively. (Table S4B).

### 3.7 Modified IFATS/ISCT index score for the measurement of adipose tissue‐derived stromal vascular fraction

The mean modified IFATS/ISCT index score was 2.5 and ranged from 1.0 to 4.44. HyTissue Nanofat by Quintero Sierra et al. scored the highest with a total score of 4.44 out of 5 and presented the most complete characterisation.^57^ AIS by Hahn et al. and NANO2 by Lo Furno et al. score the lowest number of points for characterisation (Table 5).^38^, ^45^

**TABLE 5:** Modified IFATS index score for the measurement of adipose tissue‐derived SVF.

Studies	Viability	Flow cytometry of SVF	Flow cytometry of cultured ASCs									CFU‐F	Functional assays			Total score
			CD13	CD29	CD31	CD44	CD45	CD73	CD90	CD105	CD235a		Adipogenic	Osteogenic	Chondrogenic	
Brown et al., 2017	1	1										1				3.00
Busato et al., 2020	1	0		1/9		1/9	1/9	1/9	1/9	1/9		1	1/3	1/3	1/3	3.67
Cicione et al., 2023	0	0					1/9	1/9	1/9	1/9		0	1/3	1/3		1.11
Chaput et al., 2016	1	1										1	1/3	1/3	1/3	4.00
Cohen et al., 2019	1	1										0	1/3			2.33
Van Dongen et al., 2020	1	1										1				3.00
De Francesco et al., 2018	1	0		1/9	1/9		1/9	1/9	1/9	1/9		0				1.67
François et al., 2020	1	1										1				3.00
Girard et al., 2022	1	1										1				3.00
Hahn et al., 2018	1	0										0				1.00
Hayashi et al., 2021	1	1														2.00
Lo Furno et al., 2017	1	0				1/9	1/9		1/9	1/9		0				1.44
Nürnberger et al., 2019	1	1										0	1/3	1/3	1/3	3.00
Quintera Sierra et al., 2023	1	1		1/9			1/9	1/9		1/9		1	1/3	1/3	1/3	4.44
Rodriguez et al., 2017	1	0					1/9	1/9	1/9	1/9		1	1/3	1/3	1/3	3.44
Ramaut et al., 2023	1	1										0				2.00
Raposio et al., 2017	1	1										0				2.00
Solodeev et al., 2023	1	1		1/9	1/9		1/9	1/9		1/9		0	1/3	1/3		3.22
Senesi et al., 2019	1	0			1/9		1/9		1/9		1/9	0	1/3	1/3	1/3	2.44
Sese et al., 2019	1	0										0				1.00
Tiryaki et al., 2020	1	1										0				2.00
Tiryaki et al., 2022	1	1										0	1/3			2.33
Winnier et al., 2019	1	0										1	1/3	1/3		2.67
Vezzani et al., 2018	1	1										0				2.00
Yang et al., 2021	1	1										1	1/3	1/3	1/3	4.00
Yaylaci et al., 2023	1	1										0	1/3	1/3		2.67

### 3.8 Disclosure agreements of included articles

Out of 26 studies, 8 provided a disclosure of agreement of support by the manufacturer.^33^, ^35^, ^44^, ^45^, ^47^, ^49^, ^57^, ^58^ The Nanotransfer procedure by Tulip Medical was the most used procedure in all studies.

## 4 DISCUSSION

This systematic review could not designate a mechanical or enzymatic isolation procedure of SVF as superior in terms of cell yield, viability or SVF composition. Yet, mechanical isolation procedures of tSVF is faster and easier to use in a clinical setting due less strict regulations as compared to the clinical use of enzymes.^60^, ^61^ The majority of mechanical isolation procedures are most likely less expensive than enzymatic isolation procedures due to the faster isolation of SVF which reduce the operating time.^62^ Mechanical isolation procedures can be divided roughly into four subcategories: shuffling with filtering (Nanofat procedure and modifications), shuffling and centrifugation (FAT procedure and modifications), disruption by blades or rods (i.e., Lipogem, Rigenera, Hytissue, Rotating blades) or multiple filtering steps (i.e., Lipocube). The largest difference in outcome between these types of procedures is their ability to reduce the volume of processed lipoaspirate and thus isolating tSVF as pure as possible (without adipocytes). The shuffling technique with prior centrifugation seems to be the most effective method to reduce the volume. All other mechanical methods insufficiently isolate tSVF with high number of adipocytes or lack to mention any data on volume. Hence, the superior mechanical isolation method might be centrifugation, shuffling adipose tissue forward and backward through small holes and again centrifugation.

**FIGURE 3:**
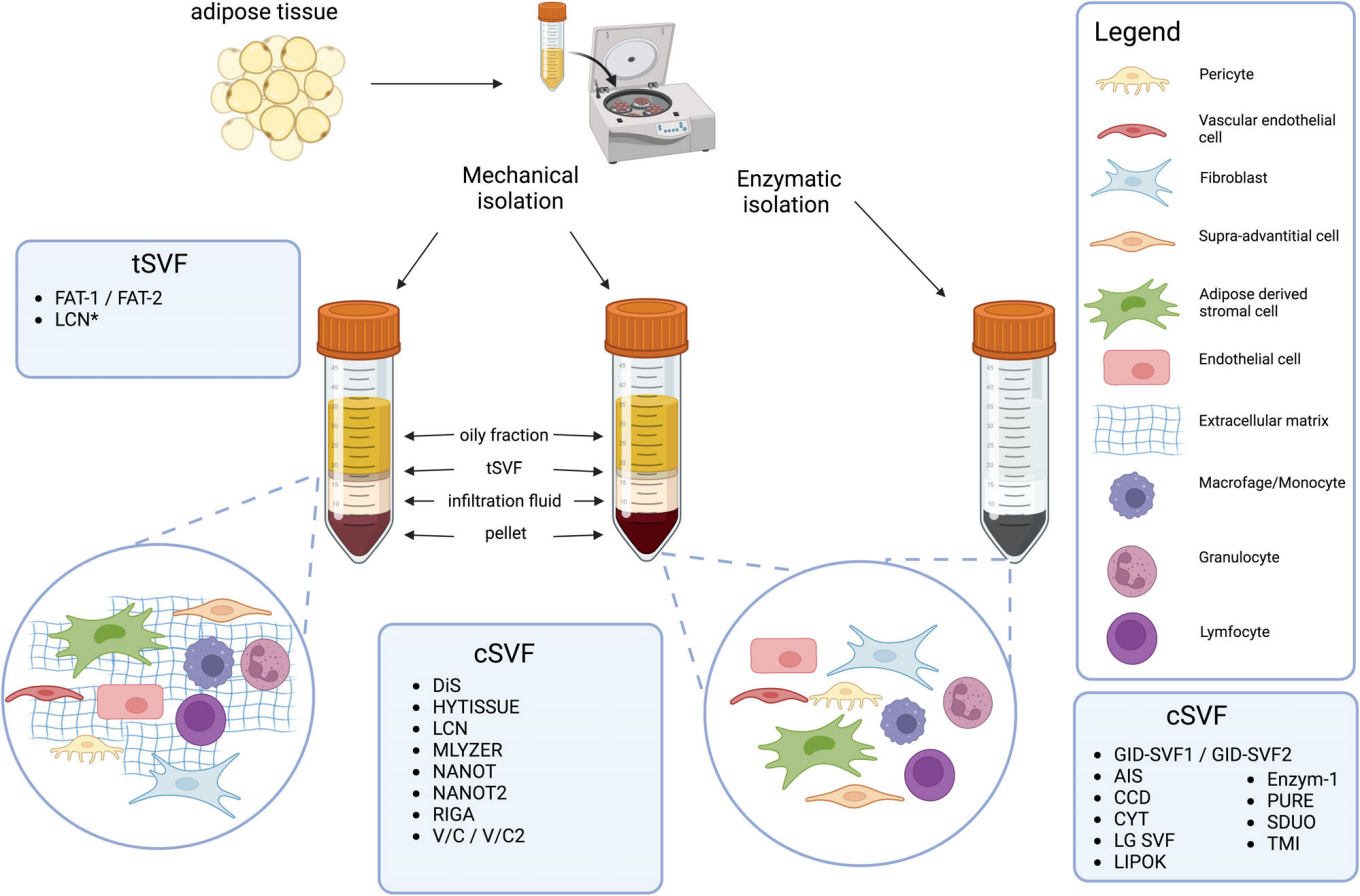
Schematic overview of different enzymatic and mechanical isolation procedures focusing on tSVF and cSVF. Only studies reporting the focused fraction were shown. AIS, automated isolation system; CCD, cell washing concentration device; CYT, Celution System Enzymatic (Cytori); DiS, dissociation by inter‐syringe processing; FAT‐1 and 2: fractionation of adipose tissue procedure, with three‐hole connector and one‐hole connector, respectively; GID SVF1 and 2, GID Europe; HYTISSUE, Hy Tissue SVF (Fidia Farmaceutici); LIPOK, Lipokit System (Medi‐khan); LGSVF, LG SVF isolation; Enzym‐1, microtissue SVF/enzymatic isolation; MLYZER, Microlyzer (T‐biotechnology); TMI, transpose RT/matrase isolation (InGeneron); NANOT, nanotranfer procedure; NANOT2, nanotransfer without filtration; LCN, LipocubeNano (Lipocube Biotech); V/C, vortexing and centrifugation; RIGA, Rigenera (HumanBrainWave); PURE, Puregraft (Eurosilicone); SDUO, stempress with duografter II (Proteal).

Enzymatic and mechanical SVF isolation procedures differ in terms of resulting volumes: mechanical isolation yields a more voluminous fraction, that is, tSVF, while enzymatic isolation yields a pellet, that is, cSVF. The voluminous aspect of tSVF is caused by preserved ECM which covers probably around 80%–90% of the volume. In contrast, cSVF is a single cell suspension without ECM (Figure 3). The choice of isolation procedure for clinical use may depend on the maximum volume possible to inject. For instance, clinical indications with small volume areas such as osteoarthritis of the carpometacarpal or temporomandibular joint allow for only a small volume tSVF or cSVF. In case of intravascular use of SVF cells, the preferred isolation procedure to be used is based on enzymes because a single suspension without ECM is warranted to prevent embolus formation. In case of intra‐articular or subcutaneous use of SVF cells, the ECM should be preserved to maximise the regenerative potential of SVF and prevent cells from migration. ECM is able to bind and release factors and cytokines to dictate cell fate, for example, migration, differentiation, proliferation or apoptosis.

The potent clinical effect of isolated tSVF and cSVF over whole lipografts is partly caused by concentration of SVF cells in a smaller volume. On the other hand, isolation procedures might also increase the regenerative potential of SVF cells caused by the application of stress, especially during mechanical isolation.^63^ Mechanical isolation uses shear stress to disrupt adipocytes. It is well known that surviving SVF cells after mechanical isolation have an up‐regulation of multipotency markers, such as CD45min/CD34pos/CD31pos/CD146pos (EPC's) and CD45min/CD31min/CD13pos, CD73pos (ADSC).^63^ This up‐regulation might result in an increased regenerative clinical effect. From this perspective, mechanical isolation procedures might not necessarily need to reduce the amount of volume of lipoaspirate and can still have an increased regenerative effect compared to unprocessed fat. These types of mechanical isolation procedures should be named differently according to their purpose: mechanical induction rather than isolation procedures.

In our previous systematic review, many SVF isolation procedures were poorly characterised and validated. Five out of thirteen included studies scored less than half of the points given for SVF characterisation. Hence, we proposed a standardised guideline based on the original IFATS and ISCT guidelines to validate SVF isolation procedures. In this systematic review, 13 out of 26 studies scored less than half of the points given which is an improvement compared to the previous review. Yet, half of the SVF isolation procedures is poorly validated. In this way, it remains difficult to compare different procedures in terms of outcome. Besides, the outcome of SVF isolation procedures in terms of cell yield and viability as well as SVF composition is influenced by many factors. It is well‐known that cell yield and SVF composition is influenced by inter‐donor variability as well as harvesting method and site. Two included studies demonstrated similar results in cell yield using the same lipoaspirates to validate two isolation protocols: LG SVF and Cytori versus Nanofat and Nanotransfer, respectively.^37^, ^53^ This is the only appropriate way to compare different SVF isolation procedures and therefore more head‐to‐head comparison studies are needed.

Seven isolation procedures included in this systematic review were analysed in our previous systematic review as well: AIS, Cytori, GID‐SVF2, Lipokit, FAT‐1 procedure, Nanofat procedure and Lipogem. Of the enzymatic isolation procedures, AIS improved in terms of speed (133 min vs. 50 min); however, cell viability decreased from 98% to 84% on average. GID‐SVF2 and Lipokit improved based on digestion efficiency because a pellet remained after enzymatic digestion rather than a voluminous fraction, which should be <5% of the initial amount of fat processed, both pellets showed a higher cell viability. Of the mechanical isolation procedures, the FAT procedure showed comparable cell yields throughout both studies. A comprehensive validation of the obtained tSVF is performed when data of both studies is combined, while validation data of the Nanofat procedure is incomplete in many studies.

The lack of standardisation of methodology, descriptive data and high heterogeneity regarding SVF characterisation is a major limitation of this systematic review and impairs proper comparisons between procedures. The use of SVF isolation procedures in clinical trials is expanding, especially in the fields of wound healing, osteoarthritis, perianal fistulas and fibrosis. For instance, Schouten et al. showed an 84% closure rate confirmed by magnetic resonance imaging in 45 patients with transsphincteric cryptoglandular fistula.^64^ In this study, the FAT procedure to isolate tSVF in combination with platelet‐rich plasma was used. Lonardi et al. showed a healing rate of 80% after treatment of diabetic foot ulcers with tSVF isolated by Lipogem as compared to a 40% closure rate after standard of care 6 months postoperative.^65^ All clinical indications with either a high inflammatory character or abundance of extracellular matrix. Paracrine factors derived from SVF act anti‐inflammatory and anti‐fibrotic and are therefore able to influence inflammatory and fibrotic processes. However, it remains unknown which type of isolation method is most suitable for which indication due to low quality validation studies as well as a lack of proper randomised clinical trials.

## 5 CONCLUSION

In terms of cell yield, viability and SVF composition, both intraoperative mechanical and enzymatic isolation procedures show comparable results. Yet, considering time, clinical feasibility and applicability, and cost, mechanical isolation procedures of SVF are favourable over enzymatic isolation procedures. Differences in clinical efficacy needs yet to be determined in head‐to‐head comparison studies between mechanical and enzymatic isolation procedures for clinical purpose. The preservation of intercellular connections as well as ECM could play a crucial role in the regenerative potential of tSVF.

## AUTHOR CONTRIBUTIONS

**M. U., N. S. and L. V.**: Analysis; interpretation of data; writing of the manuscript. **J. H. C., B. L., M. C. H. and J. A. D.**: Conceptualization; analysis; interpretation of data; revising the manuscript. All authors read and approved the final manuscript.

## FUNDING INFORMATION

The authors declare that they have no financial interests or received funding for this work.

## CONFLICT OF INTEREST STATEMENT

The authors declare that they have no competing interests or financial conflicts.

## Supporting information


**Table S1.** Specific search terms of database.

Table S2.

**Table S3.** (A) Cell yield and viability per millilitre start volume of lipoaspirate of all intraoperative enzymatic isolation procedures per study. (B) Cell yield per millilitre of end volume, viability and concentration of intraoperative mechanical isolation procedures.

**Table S4.** (A) Composition of SVF in all studies using predefined CD marker combinations. (B) Composition of SVF of all studies using different CD marker combinations.

## Data Availability

The authors confirm that the data supporting the findings of this study are available within the article and its supplementary materials.

## References

[1] Katz AJ , Llull R , Hedrick MH , Futrell JW . Emerging approaches to the tissue engineering of fat. Clin Plast Surg. 1999;26(4):587‐603. viii. 10553215

[2] Bauer‐Kreisel P , Goepferich A , Blunk T . Cell‐delivery therapeutics for adipose tissue regeneration. Adv Drug Deliv Rev. 2010;62(7–8):798‐813. 20394786 10.1016/j.addr.2010.04.003

[3] Ergün S , Tilki D , Klein D . Vascular wall as a reservoir for different types of stem and progenitor cells. Antioxid Redox Signal. 2011;15(4):981‐995. 20712422 10.1089/ars.2010.3507

[4] Zannettino AC , Paton S , Arthur A , et al. Multipotential human adipose‐derived stromal stem cells exhibit a perivascular phenotype in vitro and in vivo. J Cell Physiol. 2008;214(2):413‐421. 17654479 10.1002/jcp.21210

[5] van Dongen JA , Stevens HP , Harmsen MC , van der Lei B . Mechanical Micronization of Lipoaspirates: squeeze and emulsification techniques. Plast Reconstr Surg. 2017;139(6):1369e‐1370e. 10.1097/PRS.0000000000003372 28207564

[6] van Dongen JA , Tuin AJ , Spiekman M , Jansma J , van der Lei B , Harmsen MC . Comparison of intraoperative procedures for isolation of clinical grade stromal vascular fraction for regenerative purposes: a systematic review. J Tissue Eng Regen Med. 2018;12(1):e261‐e274. 28084666 10.1002/term.2407

[7] Ghiasloo M , Lobato RC , Díaz JM , Singh K , Verpaele A , Tonnard P . Expanding clinical indications of mechanically isolated stromal vascular fraction: a systematic review. Aesthet Surg J. 2020;40(9):NP546‐NP560. 32358957 10.1093/asj/sjaa111

[8] van Dongen JA , Stevens HP , Parvizi M , van der Lei B , Harmsen MC . The fractionation of adipose tissue procedure to obtain stromal vascular fractions for regenerative purposes. Wound Repair Regen. 2016;24(6):994‐1003. 27717133 10.1111/wrr.12482

[9] van Dongen JA , Harmsen MC , Stevens HP . Isolation of stromal vascular fraction by fractionation of adipose tissue. Methods Mol Biol. 2019;1993:91‐103. 31148081 10.1007/978-1-4939-9473-1_8

[10] Liberati A , Altman DG , Tetzlaff J , et al. The PRISMA statement for reporting systematic reviews and meta‐analyses of studies that evaluate health care interventions: explanation and elaboration. J Clin Epidemiol. 2009;62(10):e1‐e34. 19631507 10.1016/j.jclinepi.2009.06.006

[11] Schardt C , Adams MB , Owens T , Keitz S , Fontelo P . Utilization of the PICO framework to improve searching PubMed for clinical questions. BMC Med Inform Decis Mak. 2007;7:16. 17573961 10.1186/1472-6947-7-16 PMC1904193

[12] Bianchi F , Maioli M , Leonardi E , et al. A new nonenzymatic method and device to obtain a fat tissue derivative highly enriched in pericyte‐like elements by mild mechanical forces from human lipoaspirates. Cell Transplant. 2013;22(11):2063‐2077. 23051701 10.3727/096368912X657855

[13] Bourin P , Bunnell BA , Casteilla L , et al. Stromal cells from the adipose tissue‐derived stromal vascular fraction and culture expanded adipose tissue‐derived stromal/stem cells: a joint statement of the International Federation for Adipose Therapeutics and Science (IFATS) and the International Society for Cellular Therapy (ISCT). Cytotherapy. 2013;15(6):641‐648. 23570660 10.1016/j.jcyt.2013.02.006 PMC3979435

[14] Agostini F , Rossi FM , Aldinucci D , et al. Improved GMP compliant approach to manipulate lipoaspirates, to cryopreserve stromal vascular fraction, and to expand adipose stem cells in xeno‐free media. Stem Cell Res Ther. 2018;9(1):130. 29751821 10.1186/s13287-018-0886-1 PMC5948766

[15] Bellei B , Migliano E , Tedesco M , Caputo S , Picardo M . Maximizing non‐enzymatic methods for harvesting adipose‐derived stem from lipoaspirate: technical considerations and clinical implications for regenerative surgery. Sci Rep. 2017;7(1):10015. 28855688 10.1038/s41598-017-10710-6 PMC5577104

[16] Brett E , Tevlin R , McArdle A , et al. Human adipose‐derived stromal cell isolation methods and use in osteogenic and adipogenic in vivo applications. Curr Protoc Stem Cell Biol. 2017;43:2H.1.1‐2H.1.15. 10.1002/cpsc.41 PMC6487849 29140567

[17] Lee SJ , Lee CR , Kim KJ , et al. Optimal condition of isolation from an adipose tissue‐derived stromal vascular fraction for the development of automated systems. Tissue Eng Regen Med. 2020;17(2):203‐208. 31997256 10.1007/s13770-019-00238-3 PMC7105537

[18] Li Z , Mu D , Liu C , et al. The cell yields and biological characteristics of stromal/stem cells from lipoaspirate with different digestion loading ratio. Cytotechnology. 2020;72(2):203‐215. 31993890 10.1007/s10616-020-00369-9 PMC7193004

[19] Lu P , Feng C , Liang M , et al. Separation of adipose‐derived stromal vascular fraction cells by a variety of physical methods: a comparative study. Chin J Tissue Eng Res. 2020;24(13):1976‐1982.

[20] Tevlin R , McArdle A , Brett E , et al. A novel method of human adipose‐derived stem cell isolation with resultant increased cell yield. Plast Reconstr Surg. 2016;138(6):983e‐996e. 10.1097/PRS.0000000000002790 PMC8992773 27537222

[21] Alstrup T , Eijken M , Bohn AB , Møller B , Damsgaard TE . Isolation of adipose tissue‐derived stem cells: enzymatic digestion in combination with mechanical distortion to increase adipose tissue‐derived stem cell yield from human aspirated fat. Curr Protoc Stem Cell Biol. 2019;48(1):e68. 30365239 10.1002/cpsc.68

[22] Sherman LS , Condé‐Green A , Naaldijk Y , Lee ES , Rameshwar P . An enzyme‐free method for isolation and expansion of human adipose‐derived mesenchymal stem cells. J Vis Exp. 2019;154. 10.3791/59419 31885369

[23] Schmitz C , Alt C , Azares AR , et al. The composition of adipose‐derived regenerative cells isolated from lipoaspirate using a point of care system does not depend on the subject's individual age, sex, body mass index and ethnicity. Cells. 2022;12(1):30. 36611823 10.3390/cells12010030 PMC9818477

[24] Guiotto M , Raffoul W , Hart AM , Riehle MO , di Summa PG . Human platelet lysate to substitute fetal bovine serum in hMSC expansion for translational applications: a systematic review. J Transl Med. 2020;18(1):351. 32933520 10.1186/s12967-020-02489-4 PMC7493356

[25] Estrada‐Gutierrez G , Bravo‐Flores E , Ortega‐Castillo V , et al. Isolation of viable adipocytes and stromal vascular fraction from human visceral adipose tissue suitable for RNA analysis and macrophage phenotyping. J Vis Exp. 2020;164. 10.3791/61884 33191941

[26] Wang JM , Gu Y , Pan CJ , Yin LR . Isolation, culture and identification of human adipose‐derived stem cells. Exp Ther Med. 2017;13(3):1039‐1043. 28450938 10.3892/etm.2017.4069 PMC5403475

[27] Cihantimur B , Moret G , Ünal G . Fat juice: a novel approach on the usage and preparation of adipose tissue by‐products. Aesthet Surg J. 2023;43(1):Np49‐Np55. 35980950 10.1093/asj/sjac226

[28] Li J , Curley JL , Floyd ZE , Wu X , Halvorsen YDC , Gimble JM . Isolation of human adipose‐derived stem cells from lipoaspirates. Methods Mol Biol. 2018;1773:155‐165. 29687388 10.1007/978-1-4939-7799-4_13

[29] Liew LJ , Ong HT , Dilley RJ . Isolation and culture of adipose‐derived stromal cells from subcutaneous fat. Methods Mol Biol. 2017;1627:193‐203. 28836202 10.1007/978-1-4939-7113-8_12

[30] Jiang W , Cai J , Guan J , et al. Characterized the adipogenic capacity of adipose‐derived stem cell, extracellular matrix, and microenvironment with fat components grafting. Front Cell Dev Biol. 2021;9:9. 10.3389/fcell.2021.723057 PMC8489879 34616732

[31] Gentile P , Calabrese C , De Angelis B , Pizzicannella J , Kothari A , Garcovich S . Impact of the different preparation methods to obtain human adipose‐derived stromal vascular fraction cells (AD‐SVFs) and human adipose‐derived mesenchymal stem cells (AD‐MSCs): enzymatic digestion versus mechanical centrifugation. Int J Mol Sci. 2019;20(21):5471. 31684107 10.3390/ijms20215471 PMC6862236

[32] Mashiko T , Wu SH , Feng J , et al. Mechanical micronization of lipoaspirates: squeeze and emulsification techniques. Plast Reconstr Surg. 2017;139(1):79‐90. 27627056 10.1097/PRS.0000000000002920

[33] Brown JC , Shang H , Li Y , Yang N , Patel N , Katz AJ . Isolation of adipose‐derived stromal vascular fraction cells using a novel point‐of‐care device: cell characterization and review of the literature. Tissue Eng Part C Methods. 2017;23(3):125‐135. 28177263 10.1089/ten.TEC.2016.0377

[34] Chaput B , Bertheuil N , Escubes M , et al. Mechanically isolated stromal vascular fraction provides a valid and useful collagenase‐free alternative technique: a comparative study. Plast Reconstr Surg. 2016;138(4):807‐819. 27307342 10.1097/PRS.0000000000002494

[35] Cohen SR , Tiryaki T , Womack HA , Canikyan S , Schlaudraff KU , Scheflan M . Cellular optimization of nanofat: comparison of two nanofat processing devices in terms of cell count and viability. Aesthet Surg J. 2019;1(4). 10.1093/asjof/ojz028 PMC7780476 33791619

[36] De Francesco F , Mannucci S , Conti G , Dai Prè E , Sbarbati A , Riccio M . A non‐enzymatic method to obtain a fat tissue derivative highly enriched in adipose stem cells (ASCs) from human Lipoaspirates: preliminary results. Int J Mol Sci. 2018;19(7):2061. 30011969 10.3390/ijms19072061 PMC6073668

[37] François P , Giraudo L , Veran J , et al. Development and validation of a fully GMP‐compliant process for manufacturing stromal vascular fraction: a cost‐effective alternative to automated methods. Cells. 2020;9(10):2158. 32987708 10.3390/cells9102158 PMC7598595

[38] Hahn HM , Jeong KS , Yoo BY , Park JH , Jung HJ , Lee IJ . Effect of the bowl structure in an automated cell‐isolation device on stromal vascular fraction's isolation yield. J Med Devices. 2018;12(4):044501.

[39] Lo Furno D , Tamburino S , Mannino G , et al. Nanofat 2.0: experimental evidence for a fat grafting rich in mesenchymal stem cells. Physiol Res. 2017;66(4):663‐671. 28406706 10.33549/physiolres.933451

[40] Nürnberger S , Lindner C , Maier J , et al. Adipose‐tissue‐derived therapeutic cells in their natural environment as an autologous cell therapy strategy: the microtissue‐stromal vascular fraction. Eur Cell Mater. 2019;37:113‐133. 30793275 10.22203/eCM.v037a08

[41] Raposio E , Simonacci F , Perrotta RE . Adipose‐derived stem cells: comparison between two methods of isolation for clinical applications. Ann Med Surg. 2017;20:87‐91. 10.1016/j.amsu.2017.07.018 PMC5508488 28736612

[42] Rodriguez J , Pratta AS , Abbassi N , et al. Evaluation of three devices for the isolation of the stromal vascular fraction from adipose tissue and for ASC culture: a comparative study. Stem Cells Int. 2017;2017:1‐14. 10.1155/2017/9289213 PMC5340940 28321259

[43] Senesi L , De Francesco F , Farinelli L , et al. Mechanical and enzymatic procedures to isolate the stromal vascular fraction from adipose tissue: preliminary results. Front Cell Dev Biol. 2019;7:7. 31231649 10.3389/fcell.2019.00088 PMC6565890

[44] Winnier GE , Valenzuela N , Peters‐Hall J , Kellner J , Alt C , Alt EU . Isolation of adipose tissue derived regenerative cells from human subcutaneous tissue with or without the use of an enzymatic reagent. PLoS One. 2019;14(9):e0221457. 31479463 10.1371/journal.pone.0221457 PMC6719836

[45] Sesé B , Sanmartín JM , Ortega B , Matas‐Palau A , Llull R . Nanofat cell aggregates: a nearly constitutive stromal cell inoculum for regenerative site‐specific therapies. Plast Reconstr Surg. 2019;144(5):1079‐1088. 31454336 10.1097/PRS.0000000000006155 PMC6818980

[46] Busato A , De Francesco F , Biswas R , et al. Simple and rapid non‐enzymatic procedure allows the isolation of structurally preserved connective tissue micro‐fragments enriched with SVF. Cells. 2020;10(1):36. 33383682 10.3390/cells10010036 PMC7824313

[47] Vezzani B , Shaw I , Lesme H , et al. Higher Pericyte content and secretory activity of microfragmented human adipose tissue compared to enzymatically derived stromal vascular fraction. Stem Cells Transl Med. 2018;7(12):876‐886. 30255987 10.1002/sctm.18-0051 PMC6265639

[48] Yang Z , Jin S , He Y , Zhang X , Han X , Li F . Comparison of microfat, nanofat, and extracellular matrix/stromal vascular fraction gel for skin rejuvenation: basic research and clinical applications. Aesthet Surg J. 2021;41(11):NP1557‐NP1570. 33507247 10.1093/asj/sjab033

[49] Tiryaki KT , Cohen S , Kocak P , Canikyan Turkay S , Hewett S . In‐vitro comparative examination of the effect of stromal vascular fraction isolated by mechanical and enzymatic methods on wound healing. Aesthet Surg J. 2020;40(11):1232‐1240. 32514571 10.1093/asj/sjaa154

[50] Van Dongen JA , Gostelie OFE , Vonk LA , et al. Fractionation of adipose tissue procedure with a disposable one‐hole fractionator. Aesthet Surg J. 2020;40(4):NP194‐NP201. 31402379 10.1093/asj/sjz223

[51] Yaylacı S , Kaçaroğlu D , Hürkal Ö , Ulaşlı AM . An enzyme‐free technique enables the isolation of a large number of adipose‐derived stem cells at the bedside. Sci Rep. 2023;13(1):8005. 37198228 10.1038/s41598-023-34915-0 PMC10192379

[52] Girard P , Dulong J , Duisit J , et al. Modified nanofat grafting: stromal vascular fraction simple and efficient mechanical isolation technique and perspectives in clinical recellularization applications. Front Bioeng Biotechnol. 2022;10:895735. 36177178 10.3389/fbioe.2022.895735 PMC9513316

[53] Ramaut L , Moonen L , Laeremans T , Aerts JL , Geeroms M , Hamdi M . Push‐through filtration of emulsified adipose tissue over a 500‐μm mesh significantly reduces the amount of stromal vascular fraction and mesenchymal stem cells. Aesthet Surg J. 2023;43(9):Np696‐Np703. 37130047 10.1093/asj/sjad125

[54] Hayashi S , Yagi R , Taniguchi S , et al. A novel method for processing adipose‐derived stromal stem cells using a closed cell washing concentration device with a hollow fiber membrane module. Biomed Microdevices. 2021;23(1):3. 33404966 10.1007/s10544-020-00541-0 PMC7788025

[55] Solodeev I , Meilik B , Gur E , Shani N . A closed‐system technology for mechanical isolation of high quantities of stromal vascular fraction from fat for immediate clinical use. Plast Reconstr Surg. 2023;11(6):e5096. 10.1097/GOX.0000000000005096 PMC10287119 37361510

[56] Cicione C , Vadalà G , Di Giacomo G , et al. Micro‐fragmented and nanofat adipose tissue derivatives: in vitro qualitative and quantitative analysis. Front Bioeng Biotechnol. 2023;11:911600. 36733959 10.3389/fbioe.2023.911600 PMC9887143

[57] Quintero Sierra LA , Biswas R , Conti A , et al. Highly pluripotent adipose‐derived stem cell‐enriched nanofat: a novel translational system in stem cell therapy. Cell Transplant. 2023;32:9636897231175968. 37243545 10.1177/09636897231175968 PMC10226300

[58] Tiryaki T , Cohen SR , Canikyan Turkay S , et al. Hybrid stromal vascular fraction (Hybrid‐SVF): a new paradigm in mechanical regenerative cell processing. Plast Reconstr Surg. 2022;10(12):e4702. 10.1097/GOX.0000000000004702 PMC9803457 36601591

[59] Raposio E , Bertozzi N . How to isolate a ready‐to‐use adipose‐derived stem cells pellet for clinical application. Eur Rev Med Pharmacol Sci. 2017;21(18):4252‐4260. 29028071

[60] Raposio E , Ciliberti R . Clinical use of adipose‐derived stem cells: European legislative issues. Ann Med Surg. 2017;10(24):61‐64. 10.1016/j.amsu.2017.11.002 PMC5709339 29204274

[61] Turner LG . Federal regulatory oversight of US clinics marketing adipose‐derived autologous stem cell interventions: insights from 3 new FDA draft guidance documents. Mayo Clin Proc. 2015;90(5):567‐571. 25939934 10.1016/j.mayocp.2015.02.003

[62] Childers CP , Maggard‐Gibbons M . Understanding costs of care in the operating room. JAMA Surg. 2018;153(4):e176233. doi:10.1001/jamasurg.2017.6233 29490366 PMC5875376

[63] Banyard DA , Sarantopoulos CN , Borovikova AA , et al. Phenotypic analysis of stromal vascular fraction after mechanical shear reveals stress‐induced progenitor populations. Plast Reconstr Surg. 2016;138(2):237e‐247e. 10.1097/PRS.0000000000002356 PMC5845841 27465185

[64] Schouten WR , Arkenbosch JHC , van der Woude CJ , et al. Efficacy and safety of autologous adipose‐derived stromal vascular fraction enriched with platelet‐rich plasma in flap repair of trans‐sphincteric cryptoglandular fistulas. Tech Coloproctol. 2021;25(12):1301‐1309. 34606026 10.1007/s10151-021-02524-6 PMC8580893

[65] Lonardi R , Leone N , Gennai S , Trevisi Borsari G , Covic T , Silingardi R . Autologous micro‐fragmented adipose tissue for the treatment of diabetic foot minor amputations: a randomized controlled single‐center clinical trial (MiFrAADiF). Stem Cell Res Ther. 2019;10(1):223. 31358046 10.1186/s13287-019-1328-4 PMC6664586

