# Supplementary material for: Comparing mechanical and enzymatic isolation procedures to isolate adipose‐derived stromal vascular fraction: A systematic review
Source: Wound Repair Regen. 2024 Oct 24;32(6):1008–21. doi: 10.1111/wrr.13228 (PMC11584359; doi:10.1111/wrr.13228)
Supplement: Supplementary file 1 — Table S1. Specific search terms of database. [file WRR-32-1008-s003.docx]

|  |
| --- |
| **Search terms Pubmed:**  ((((Adipose Tissue [Mesh] OR Adipocytes [Mesh] OR Fat [tiab] OR Lipoaspirate* [tiab])) AND (Cell separation [Mesh] OR Isolat* [tiab] OR Dissociat* [tiab] OR Emulsification [tiab] OR  Concentrat* [tiab] OR Digest* [tiab] OR Obtained [tiab])) AND (Stem cells [Mesh] OR Stromal cells [Mesh] OR Autologous progenitor cell* [tiab] OR Stromal vascular* [tiab] OR  Regenerative cell* [tiab] OR Vascular stroma [tiab]))  Restriction: Only human |
| **Search terms Embase:**  (’adipose tissue’:ab,ti OR ’adipocytes’:ab,ti OR ’fat’:ab,ti OR lipoaspirate*:ab,ti AND (’cell separation’ OR isolat*:ab,ti OR dissociat*:ab,ti OR ’emulsification’:ab,ti OR concentrat*:ab,ti  OR digest*:ab,ti OR ’obtained’:ab,ti) AND (’stem cells’:ab,ti OR ’stromal cells’:ab,ti OR ’autologous progenitor cell’:ab,ti OR ’autologous progenitor cells’:ab,ti OR ’stromal vascular’:ab,ti  OR ’stromal vascular fraction’:ab,ti OR ’regenerative cell’:ab,ti OR ’regenerative cells’:ab,ti OR ’vascular stroma’:ab,ti)) AND [embase]/lim NOT [medline]/lim AND ’article’/it  Restriction: Only EMBASE  **Search term Cochrane library:** |
| (adipose tissue OR adipocytes OR fat OR lipoaspirate*) AND (cell separation OR Isolat* OR Dissociat* OR Emulsification OR Concentrat* OR Digest* OR Obtained) AND (stem cells OR  stromal cells OR autologous progenitor cell* OR stromal vascular* OR regenerative cell* OR vascular stroma) |

Table 1. Specific search terms of database
