# Supplementary material for: Comparing mechanical and enzymatic isolation procedures to isolate adipose‐derived stromal vascular fraction: A systematic review
Source: Wound Repair Regen. 2024 Oct 24;32(6):1008–21. doi: 10.1111/wrr.13228 (PMC11584359; doi:10.1111/wrr.13228)
Supplement: Supplementary file 2 — Table S2. [file WRR-32-1008-s002.docx]

| **Name** | **Author** | **Total patients (n)** | **Female (n)** | **Age mean +SD(y)** | **Age variance (y)** | **BMI mean +sd (kg)** | **BMI range (kg)** | **Liposuction** | **Donor site** | **Infiltration (1/0)** | **Cannula (mm)** | **Pressure (yes/no)** |
| --- | --- | --- | --- | --- | --- | --- | --- | --- | --- | --- | --- | --- |
| GIDSVF1 | Brown et al. 2017 | 23 | 21 | 44 | 21-70 | 28 | 22-37 | TL | A | 1 | NR | NR |
| GIDSVF2 |  | 4 | 4 | 52 | 32-68 | 26.6 | 22-34 |  |  |  |  |  |
| HYTISSUE | Busato et al. 2020 | 27 | 27 | NR | 41-69 | NR | NR | TL | A | 1 | 11G 6  holes | NR |
| NANO | Cicione et al. 2023 | 18 | 9 | 63 | 52-74 | NR | NR | TL | A | 1 | NR | NR |
| LIPOG |  |  |  |  |  |  |  |  |  |  |  |  |
| V/C | Chaput et al. 2016 | 21 | NR | 38 ±9.9 | NR | 26.5±2.5 | NR | L | A | 0 | 4, blunt tip | Yes, 400mmHg |
| DiS |  |  |  |  |  |  |  |  |  |  |  |  |
| LCN | Cohen et al. 2019 | 10 | 10 | 47 | 33-54 | 27 | 19-34 | TL | NR | 1 | 2.4 | NR |
| NANOT |  |  |  |  |  |  |  |  |  |  |  |  |
| FAT-1 | Van Dongen et al. 2020 | 3 | NR | NR | NR | NR | NR | L | NR | 1 | Sorensen cannula | No |
| FAT-2 |  |  |  |  |  |  |  |  |  |  |  |  |
| RIGA | De Francesco et al. 2018 | 4 | 4 | NR | 28-50 | NR | NR | L | NR | 1 | 38 | Yes, 500mbar |
| CYT | François et al. 2020 | 33 | NR | NR | NR | NR | NR | L | NR | 1 | 27 | NR |
| LGSVF |  | 6 |  |  |  |  |  |  |  |  |  |  |
| SHUF20 | Girard et al. 2022 | 9 | 9 | 43 | 30-56 | 26.4 | 23.2-29.6 | L | NR | 0 | 4 | Yes, 400mmHg |
| SHUF30 |  |  |  |  |  |  |  |  |  |  |  |  |
| SHUF40 |  |  |  |  |  |  |  |  |  |  |  |  |
| AIS | Hahn et al. 2018 | 15 | 12 | 32 ±3.1 | 26-40 | 24.7±3.6 | NR | TL | A + T | 1 | 3 with 1.5mm side holes | Yes |
| CCD | Hayashi et al. 2021 | 14 | 13 | NR | NR | NR | NR | TL | A + T | 1 | 3-4 blunt tip | NR |
| NANO | Lo Furno et al. 2017 | 8 | NR | 40 | 30-60 | NR | NR | NR | A | 0 | 3 with 1mm side holes | NR |
| NANO2 |  |  |  |  |  |  |  |  |  |  |  |  |
| Enzym-1 | Nürnberger et al. 2019. | 23 | NR | 39 ±10 | NR | 33.6±6.7 | NR | TL | NR | 0 | NR | NR |
| HT-NANO | Quintero Sierra et al. 2023 | 8 | 8 | NR | 41-69 | NR | NR | TL | A | 1 | 11G, 6 holes | No |
| GIDSVF1 | Rodriguez et al. 2017 | 4 | NR | 37 ±5.1 | 22-49 | 36.8±5.1 | 25-38 | TL | A | 0 | 3, blunt tip | Yes, 300mmHg |
| PURE |  |  |  |  |  |  |  |  |  |  |  |  |
| SDUO |  |  |  |  |  |  |  |  |  |  |  |  |
| NANOT | Ramaut et al. 2023 | 13 | 13 | 39 | 28-49 | 24.2 | 21-26 | TL | A | 1 | 3, blunt tup | No |
| NANOT2 |  |  |  |  |  |  |  |  |  |  |  |  |
| RIGA | Senesi et al. 2019 | 5 | 3 | 54 ±10 | 45-65 | NR | NR | L | NR | 1 | NR | NR |
| LIPOG |  |  |  |  |  |  |  |  |  |  |  |  |
| GIDSVF1 | Sese et al. 2019 | 20 | NR | NR | NR | NR | NR | L | A | 1 | NR | NR |
| NANOT |  | 6 | NR | NR | NR | NR | NR | TL | A | 1 | Carraway Harvester | NR |
| RBs | Solodeev et al. 2023 | 30 | NR | 42±9.4 | NR | 27.3±3.5 | NR | TL | NR | 1 | 3, blunt tip | Yes |
| LCN | Tiryaki et al. 2020 | 10 | 10 | 39 | 26-52 | 26 | 17-35 | L | T | 1 | 2 multihole | NR |
| LCN | Tiryaki et al. 2022 | 11 | NR | NR | NR | NR | NR | TL | A + T + LB | 1 | 2.4 | NR |
| TMI | Winnier et al. 2019 | 12 | 12 | 40 | 32-59 | NR | NR | NR | NR | 0 | NR | NR |
| LIPOK | Raposio et al. 2017 | NR | NR | NR | NR | NR | NR | L | NR | 0 | 4 | NR |
| LIPOG | Vezzani et al 2018 | NR | NR | NR | 26-71 | NR | NR | TL | A | 1 | 13G | Yes |
| MLYZER | Yaylaci et al. 2023 | 10 | 6 | NR | 30-55 | 29 | 25-35 | L | NR | 0 | NR | NR |
| NANOT | Yang et al. 2021 | 12 | 10 | 40 | 32-55 | 24.5 | 19.5-27.2 | TL | A + T | 1 | 2,5 | No |
| SVFG |  |  |  |  |  |  |  |  |  |  |  |  |

Table 2. Study characteristics

AIS Automated Isolation System; CCD = Cell washing Concentration Device, CYT Celution System Enzymatic (Cytori); DiS = Dissociation by inter-Syringe processing ;FAT-1 and 2: Fractionation of adipose tissue procedure, with three-hole connector and one-hole connector respectively GID SVF1 and 2 (GID Europe); HT-NANO Hy-tissue Nanofat (Fidia Farmaceutici; HYTISSUE Hy Tissue SVF (Fidia Farmaceutici); LIPOG Lipogems (Lipogems); LIPOK Lipokit System (Medi-khan); LGSVF LG SVF isolation; Enzym-1 (microtissue SVF/enzymatic isolation); MLYZER= Microlyzer (T-biotechnology); NANO Nanofat procedure; NANO fat 2.0 procedure; NANOT Nanotranfer procedure; NANOT2 Nanotransfer without filtration; LCN LipocubeNano (Lipocube Biotech); V/C Vortexing and Centrifugation; RBs Rotating blades system RIGA Rigenera (HumanBrainWave); PURE Puregraft (Eurosilicone); SHUF20,30, and 40 = shuffling 20, 30 and 40 times respectively; SVFG SVF gel; SDUO stempress with duografter II (Proteal), TL=tumescent liposuction, L=liposuction, A=abdomen, T= thigh, F=flanks, LB= lower back, 1 = used infiltration prior to liposuction, 0 = not mentioned nor used infiltration prior to liposuction. NR= not reported
