# Supplementary material for: Comparing mechanical and enzymatic isolation procedures to isolate adipose‐derived stromal vascular fraction: A systematic review
Source: Wound Repair Regen. 2024 Oct 24;32(6):1008–21. doi: 10.1111/wrr.13228 (PMC11584359; doi:10.1111/wrr.13228)
Supplement: Supplementary file 3 — Table S3. (A) Cell yield and viability per millilitre start volume of lipoaspirate of all intraoperative enzymatic isolation procedures per study. (B) Cell yield per millilitre of end volume, viability and concentration of intraoperative mechanical isolation procedures. [file WRR-32-1008-s004.docx]

| **Enzymatic isolation procedure** | **Cell yield x10^5^ cells/ml** | **SD** | **Viability nucleated cells (%)** | **SD** |
| --- | --- | --- | --- | --- |
| AIS (Hahn, 2018) | 2,3 | - | 84 | - |
| CCD (Hayashi, 2021) | 5,2 | 1.7 | 75 | 2.4 |
| CYT (Francois, 2020) | 2,5 | 1.2 | 87 | 4 |
| GID SVF1 (Brown, 2017) | 9,6 | 2.1 | 87 | 1,9 |
| GID SVF1 (Sese, 2019) | 6,6 | 0,5 | 77 | - |
| GID SVF1 (Rodriguez, 2017) | 4,3 | 0,4 | 82 | 1,4 |
| GID SVF2 (Brown, 2017) | 10,1 | 0.1 | 82 | 1,4 |
| LGSVF (Francois, 2020) | 4,2 | 4.2 | 93 | 3,5 |
| LIPOK (Raposio, 2017) | 3,5 | - | 99 | - |
| Enzym-1 (Nürnberger, 2019)^a^ | 5,3* | - | 85* | - |
| PURE (Rodriguez, 2017) | 2,5 | 0,7 | 77 | 1,1 |
| SDUO (Rodriguez, 2017) | 5,3 | 2,1 | 70 | 2,4 |
| TMI (Winnier, 2018)^a^ | 7,2 | 0,9 | 86 | 2,2 |

Tabel 3A Cell yield and viability per milliliter start volume of lipoaspirate of all intraoperative enzymatic isolation procedures per study

*Significantly best procedure tested in their study (p<0.05); ^a^ No exact data mentioned in text, data extracted from figures by author M.U. AIS Automated Isolation System (Cellunit); CCD cell washing concentration device; CYT Celution System Enzymatic (Cytori); GID SVF1 and 2 (GID Europe); LGSVF LG SVF isolation protocol; LIPOK Lipokit System (Medi-khan); Enzym-1 (enzymatic isolation); PURE Puregraft (Eurosilicone), SDUO stempress with duografter II (Proteal); TMI transpose RT/matrase isolation (InGeneron)

| **Mechanical isolation procedure** | **Cell yield x10^5^ cells/ml** | **SD** | **Viability nucleated cells (%)** | **SD** | **Start volume (ml)** | **End volume (ml)** | **End as % of start volume** |  |
| --- | --- | --- | --- | --- | --- | --- | --- | --- |
| HYTISSUE (Busato, 2020) | 0.41 | 0,2 | NR | - | 30 | P | NR |  |
| LIPOG (Cicione, 2023) | NR | - | NR | - | 50 | NR | NR |  |
| NANO (Cicione, 2023) | NR | - | NR | - | 10 | NR | NR |  |
| NANOT (Cohen, 2019) | 14.4 | - | 96.1 | - | 10 | NR | NR |  |
| LCN (Cohen, 2019) | 22.4 | - | 96.1 | - | 20 | NR | NR |  |
| DiS (Chaput,2016)^a^ | 0.80 | - | 45.5 | 3.5 | NR | P | NR |  |
| V/C (Chaput, 2016)^a^ | 0.46 | - | 54.5 | 7.5 | NR | P | NR |  |
| FAT-1 (van Dongen, 2020) | 23.5 | 2.9 | NR | - | 10 | 1.1 | 11 |  |
| FAT-2 (van Dongen, 2020) | 26.7 | 4.6 | NR | - | 10 | 1.1 | 11 |  |
| RIGA30 (De Francesco, 2018) | NR | - | NR | - | NR | NR | NR |  |
| RIGA45 (De Francesco, 2018) | NR | - | NR | - | NR | NR | NR |  |
| NANO (Lo Furno, 2017) | NR | - | NR | - | NR | NR | NR |  |
| NANO2 (Lo Furno, 2017) | NR | - | NR | - | NR | NR | NR |  |
| SHUF20 (Girard, 2022) | 3,7 | - | NR | - | 10 | NR | NR |  |
| SHUF30 (Girard, 2022) | 2,9 | - | NR | - | 10 | NR | NR |  |
| SHUF40 (Girard, 2022) | 2,1 | - | NR | - | 10 | NR | NR |  |
| HT-NANO (Quintero Sierra et al,2023) | 0.37 | 1.3 | NR | - | 10 | NR | NR |  |
| RBs (Solodeev, 2023) | 2.01 | 1.2 | NR | - | NR | NR | NR |  |
| LIPOG (Senesi, 2019) | NR | - | NR | - | NR | 10 | NR |  |
| RIGA (Senesi, 2019 | NR | - | NR | - | NR | 10 | NR |  |
| NANOT (Sese, 2019) | 6,6 | - | 76,8 | - | 20 | NR | NR |  |
| LCN (Tiryaki, 2020) | 9,6 | - | 97,5 | - | 20 | NR | NR |  |
| LCN (Tiryaki, 2022) | 19 | 0,1 | NR | - | 20 | NR | NR |  |
| NANOT (Ramaut, 2023) | 12,4 | 1.1 | 93,2 | 0,98 | 10 | NR | NR |  |
| NANOT2 (Ramaut, 2023) | 17,2 | 1.4 | 91,4 | 0,6 | 10 | NR | NR |  |
| V/C2 (Raposio, 2017) | 1,25 | - | 99 | - | 80 | NR | NR |  |
| LIPOG (Vezzani, 2018) | 0,03 | - | NR | - | 60 | 20-30 | 33-50 |  |
| NANOT (Yang 2021)^a^ | 0,42 | 0,1 | NR | - | 10 | 8,2 | 82 |  |
| SVFG (Yang, 2021)^a^ | 4,5 | 1,0 | NR | - | 10 | 2,0 | 20 |  |
| MLYZER (Yaylaci,2023)^a^ | 20 | - | 90 | - | 10 | 7,9 | 12 |  |

Table 3B: Cell yield per milliliter of end volume, viability and concentration of intraoperative mechanical isolation procedures.

^a^ No exact data mentioned in text, data extracted from figures by author MU. DiS = Dissociation by inter-Syringe processing; FAT-1 and 2 = Fractionation of Adipose Tissue procedure with three-hole connector and one-hole connector respectively; HT-NANO = HyTissue Nanofat; HYTISSUE = Hytissue; LCN = LipoCubeNano; LIPOG = Lipogem; MLYZER = Microlyzer (T-biotechnology); NANO = Nanofat procedure; NANOT = NanoTransfer; NANO2 = Nanofat 2.0 procedure; NANOT2 = Nanotransfer without filtration; RBs = Rotating blades system; RIGA45 = Rigenera 45s; RIGA30 = Rigenera 30s; RIGA = Rigenera; SHUF20,30, and 40 = shuffling 20, 30 and 40 times respectively; SVFG = SVF gel; V/C vortexing and centrifugation; V/C2 = vortexing and centrifugation 2. NR= not reported
