# Supplementary material for: Comparing mechanical and enzymatic isolation procedures to isolate adipose‐derived stromal vascular fraction: A systematic review
Source: Wound Repair Regen. 2024 Oct 24;32(6):1008–21. doi: 10.1111/wrr.13228 (PMC11584359; doi:10.1111/wrr.13228)
Supplement: Supplementary file 4 — Table S4. (A) Composition of SVF in all studies using predefined CD marker combinations. (B) Composition of SVF of all studies using different CD marker combinations. [file WRR-32-1008-s001.docx]

|  | Enzymatic isolation procedures | | | | | | | Mechanical isolation procedures | | | | | | |  |  |
| --- | --- | --- | --- | --- | --- | --- | --- | --- | --- | --- | --- | --- | --- | --- | --- | --- |
|  |  | GIDSVF1(Brown 2017) | | GIDSVF2(Brown 2017) | CYT (Francois, 2018) | CCD (Hayashi, 2021) | LGSVF (Francois, 2018) | Enzym-1 (Nürnberger,2019) | FAT-1 (van Dongen,2020) | | FAT-2 (van Dongen,2020) | NANO (Chaput2016) | V/C (Chaput,2016) | RBs (Solodeev, 2023) | NANO (Yang, 2021) | SVFG (Yang,2021) |
| Adipose derived cells (CD45-) | Vascular endothelial/Endothelial progenitor cells (CD31+/CD34+) | 9.4% | | 7.3% |  | 11,6% |  | 39% | 12.0% | | 19.3% | 0.2% | 1.6% | 3% |  |  |
|  | Endothelial cells (CD31+/CD34-) |  | |  |  |  |  |  |  | |  |  |  |  |  |  |
|  | Pericytes (CD31-/CD146+) (CD31-/CD34-/+)(CD34-/CD146+/CD90+) | 31.3% | | 18% | 12.7% |  | 10,3% | 19% | 0.3% | | 0.5% |  |  |  |  |  |
|  | Stromal cell population (CD31-/CD34+) | 16.3% | | 13.1% |  | 52.6% |  |  |  | |  | 38.1% | 5.8% | 22.7% | 6,5% | 5,5% |
| Blood derived cells (CD45+) | Lymphocytes (CD31+/CD34-/CD90-/CD105-/CD146-) |  | |  |  |  |  |  |  | |  |  |  |  |  |  |
|  | Leukocytes (CD31-/CD34-/CD90-/CD105-/CD146-) |  | |  |  |  |  |  | 5.3% | | 5.3% |  |  |  |  |  |
|  | Hematopoetic stem cell (CD31dim/CD34+/CD90-/CD105-/CD146-) |  | |  |  |  |  |  | 0.1% | | 0.2% |  |  |  |  |  |

Table 4A. Composition of SVF in all studies using predefined CD marker combinations

^a^ No exact data described in text, data extracted from figures by author M.U. Stromal cell population (CD31min/CD34pos) consists of supra-adventitial cells, ASCs and pericytes, only pericytes defined as CD31min/CD146pos, CD31min/CD34min/pos or CD34min/CD146pos/CD90pos are placed separately in the table. CCD Cell washing Concentration Device ;CYT Celution System Enzymatic (Cytori); FAT-1 and 2 Fractionation of adipose tissue procedure, GID SVF1 and 2 (GID Europe); LIPOG Lipogems (Lipogems);; LGSVF LG SVF isolation; Enzym-1 (microtissue SVF/enzymatic isolation); NANO Nanofat procedure; LCN LipocubeNano; RBs Rotating blades system; V/C Vortexing and Centrifugation; SVFG SVF gel

|  | (Nürnberger, 2019) | Chaput, 2016 | | Cohen, 2019 | | Francois, 2020 | | Tiryaki, 2020 | Tiryaki, 2022 | Vezzani, 2018 | Yang, 2021 | |
| --- | --- | --- | --- | --- | --- | --- | --- | --- | --- | --- | --- | --- |
| Procedure | Enzym-1 | DiS | V/C | LCN | NANO | LGSVF | CYT | LCN | LCN | LIPOG | NANO | SVFG |
| **Endothelial progenitor cells** |  |  |  |  |  |  |  |  |  |  |  |  |
| CD45-/CD31+ |  |  |  | 12% | 5.8% |  |  |  |  |  |  |  |
| CD45-/CD31+/CD34+ | 39% |  |  |  |  |  |  |  |  |  |  |  |
| **Pericytes** |  |  |  |  |  |  |  |  |  |  |  |  |
| CD90+/CD146+ (subset) | 36% |  |  |  |  |  |  |  |  |  |  |  |
| CD45-/CD34-/CD146+ |  |  |  |  |  | 12.7% | 10.3% |  |  |  |  |  |
| CD45-/CD31-/CD146+ | 19% |  |  |  |  |  |  |  |  |  |  |  |
| CD31+/CD34+/CD146+ |  |  |  |  |  |  |  |  |  |  |  |  |
| CD105+/CD73+ |  |  |  |  |  |  |  | 1.8% |  |  |  |  |
| CD146+/CD34- |  |  |  |  |  |  |  |  |  | 33.5% |  |  |
| **Adipose stromal cells** |  |  |  |  |  |  |  |  |  |  |  |  |
| CD34+/CD90+ (Subset) | 67% |  |  |  |  |  |  |  | 17.0% |  |  |  |
| CD45-/CD90+ |  |  |  | 7.9% | 3.1% |  |  |  |  |  |  |  |
| CD73+/CD105+ |  |  |  |  |  |  |  |  | 6.8% |  |  |  |
| CD73+/CD90+ |  |  |  | 37.3% | 27.9% |  |  |  |  |  |  |  |
| CD34+/CD146-/CD90+ |  |  |  |  |  | 39.5% | 43.9% |  |  |  |  |  |
| CD45-/CD90+/CD73+ |  |  |  |  |  |  |  | 2% |  |  |  |  |
| CD90+/CD44+ |  |  |  |  |  |  |  | 10.2% |  |  |  |  |
| CD45-/CD31-/CD34+ |  | 38.1% | 5.8% |  |  |  |  |  |  |  |  |  |
| **Endothelial cells** |  |  |  |  |  |  |  |  |  |  |  |  |
| CD45-/CD31+/CD34+ |  | 1.6% | 0.2% |  |  |  |  |  |  |  |  |  |
| CD45-/CD31+ |  |  |  |  |  |  |  |  |  |  | 43.4% | 39.9% |
| CD45-/CD34+/CD146+ |  |  |  |  |  | 8.4% | 7.6% |  |  |  |  |  |
| **Monocytes/macrophages** |  |  |  |  |  |  |  |  |  |  |  |  |
| CD45+/CD14+ |  |  |  | 3% | 2.4% |  |  |  |  |  |  |  |
|  |  |  |  |  |  |  |  |  |  |  |  |  |
| **Supra-advantitial cells** |  |  |  |  |  |  |  |  |  |  |  |  |
| CD34-/CD146+ |  |  |  |  |  |  |  |  |  |  |  |  |

Table 4B. Composition of SVF of all studies using different CD marker combinations

^a^ No exact data described in text, data extracted from figures by authors M.U. CYT Celution System (Cytori); DiS = Dissociation by inter-Syringe processing; LIPOG Lipogems (Lipogems); LGSVF LG SVF isolation; Enzym-1 (microtissue SVF/enzymatic isolation); NANO Nanofat procedure; LCN LipocubeNano; V/C Vortexing and Centrifugation; SVFG= SVF gel
